# Optimization of SVM Parameters with Hybrid CS-PSO Algorithms for Parkinson's Disease in LabVIEW Environment

**DOI:** 10.1155/2019/2513053

**Published:** 2019-05-02

**Authors:** Duygu Kaya

**Affiliations:** Department of Electrical-Electronics Engineering, Faculty of Engineering, Firat University, Elazig, Turkey

## Abstract

Optimization is the process of achieving the best solution for a problem. LabVIEW based on an SVM model is proposed in this paper to get the best SVM parameters using the hybrid CS and PSO method. PCA is used as a preprocessor of SVM for reducing the dimension of data and extracting features of training samples. Also, SVM parameters are optimized for Parkinson's disease data by combining CS and PSO. The designed system is used to determine the best SVM parameters, and it is compared to PSO and CS optimization methods and found that the used CS-PSO hybrid optimization method is better. The hybrid model shows that the accuracy of the performance achieved is 97.4359%. Also, the data classification results obtained by using SVM parameters determined by optimization are measured by precision, recall, *F*1 score, false positive rate (FPR), false discovery rate (FDR), false negative rate (FNR), negative predictive value (NPV), and Matthews' correlation coefficient (MCC) parameters.

## 1. Introduction

Parkinson's disease (PD) is a neurological disorder that affects the standard of life of the patients and their relatives. PD is more widespread in countries where the pretty old population is high. According to statistics, in the US, about one million people will be affected with Parkinson's disease (PD) by 2020 and more than 10 million people worldwide will be living with PD. The possibility of men with Parkinson's disease is 1.5 times higher than that of women [1]. The main symptoms of this disease are tremor-trembling, stiffness in the body, slowness of movements, and impaired balance. As the disease progresses, patients may experience difficulty in vital tasks such as walking, speech, swallowing, and chewing, emotional changes, and sleep disorders. PD symptoms occur slowly, and in some people, the disease progresses faster than others. The intensity of the symptoms varies from person to person and does not create the same effect [2]. Although there is no treatment method to eliminate this disease completely, drug treatment is applied to reduce the symptoms seen in the early stages of the disease. For diagnosis of the disease, walking and vocal analysis methods are used. Machine learning methods have been used to diagnose the disease [3]. Among supervised learning algorithms, support vector machine (SVM) is based on statistical learning theory and is the most effective algorithm for predicting performance for nonlinear problems. It has high generalization capabilities. SVM is a powerful technique for overcoming classification problems, image processing, and disease diagnosis with excellent performance.

Before the SVM is applied, a “feature transformation” operation must be performed, which is the process of transforming the data into a new set of data at a dimension that can express less features. With this, dimension of data reduces and excessive numbers of unimportant features are removed. In this study, PCA is used for dimension reduction.

In cases where the difference between the reduced dimension data is too high, the normalization process is performed to handle the data in a single order. In addition, normalization is also used which makes use of mathematical functions to move data in different scaling systems to a common system and make them comparable. In this study, the *Z*-score normalization method is used. *Z*-score is calculated by subtracting the average value from each variable value and dividing the obtained difference by the standard deviation.

Particle swarm optimization (PSO) is an optimization technique designed to deliver the best solution to the system. Also, the cuckoo search algorithm (CS) is another optimization method. It has less parameters, easy to implement, and efficient. In this paper, both of them are used for optimization of SVM parameters.

### 1.1. Motivation

Accurate and reliable diagnosis is very important for human health. In this study, different optimization algorithms have been used to obtain the best SVM parameters for predicting Parkinson's disease. The proposed hybrid CS-PSO-SVM model provided an accuracy of 97.4359% and is superior to the PSO-SVM and CS-SVM models.

### 1.2. Contribution

- A different work environment for researchers has been proposed using LabVIEW, a visual programming language instead of only text-based programming languages
- Hybrid optimization methods are used for obtaining the best SVM parameters

### 1.3. Sections

The paper is organized as follows: In Section 2, used data are described. In Section 3, PCA, SVM, PSO, and CS are explained. In Section 4, the LabVIEW programming language and information about its features are given. In Section 5, the proposed model CS-PSO-SVM is described. In Section 6, experimental results are given. The last section of the study includes discussion and conclusions.

## 2. Dataset and Features

Appropriate C value parameter is chosen [4]. The attributes of these data which are related to biomedical voice measurements are given in Table 1.

## 3. Description of Used Techniques

**Figure 1:**
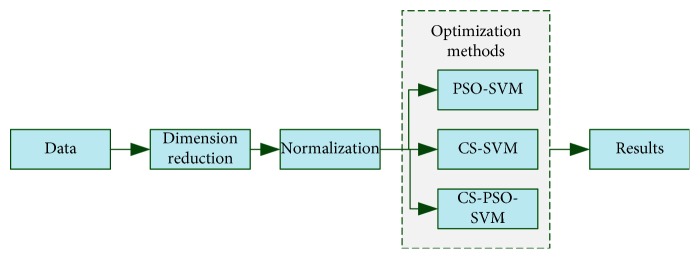
The diagram of the used techniques.

Dimension reduction and normalization procedures were performed to extract properties from the used data to ensure that they are in a single order. Then, optimization methods are applied to SVM.  Figure 1 shows the diagram of the used techniques.

### 3.1. Principal Component Analysis (PCA)

**Figure 2:**
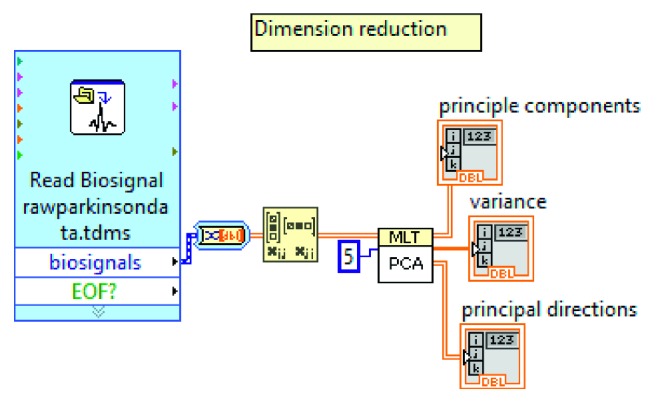
Dimension reduction program for PCA on LabVIEW.

PCA is a technique that has a wide range of uses for reducing the insignificant features of the data. The idea underlying the PCA is to represent a data plane by separating it into orthogonal axes to reflect the data in small linear combinations. In other words, PCA reduces the data dimension to extract features.  Figure 2 shows the used dimension reduction program on LabVIEW.

### 3.2. Normalization with *Z*-Score

Statistical normalization is performed to treat the data in a single order when there is a lot of difference between the data. Also, another objective is to use mathematical functions to translate data from different systems into a common system and make them comparable. In the *Z*-score normalization, the numbers are normalized to the distance of the mean value. In addition, dividing by standard deviation, the mobility between numbers (rate of change) normalizes the distance to the average. In other words, the mean and standard deviation values are taken into account. Standard deviation, mean, and *Z*-score are calculated by using the following equations, respectively:

(1) $$\begin{array}{c} s=\sqrt{\underset{i}{\sum }\frac{{\left(x_{i}-\bar{x}\right)}^{2}}{n}},\\ \bar{x}=\frac{\sum_{i}x_{i}}{n},\\ z=\frac{x_{i}-\bar{x}}{s}. \end{array}$$

### 3.3. Support Vector Machine (SVM)

**Figure 3:**
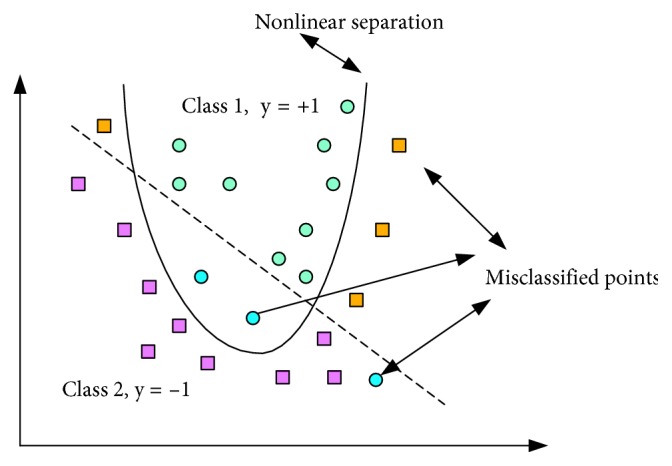
Nonlinear SVM.

**Figure 4:**
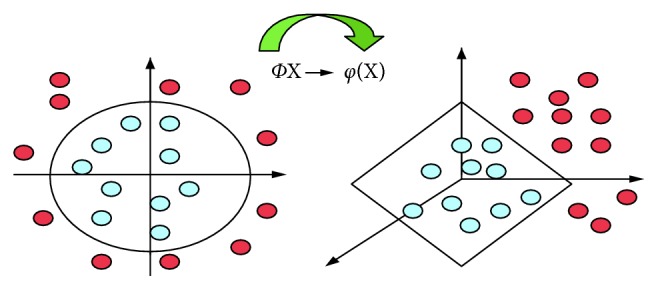
Transfer of input data to the property plane.

SVM is an important tool for machine learning (ML) derived from statistical learning theory [5–8]. SVM is one of the most used algorithms for classification and regression tasks because it has high performance and generalization capability [9–14]. The main idea behind SVM is to obtain a linear discriminatory function that separates classes from each other by a hyperplane. SVM finds the hyperplane which maximizes the margin between samples and the class boundary for linearly separable data, but for real-world applications, as shown in Figure 3, the nonlinear transformations with the kernel functions are essential to move the datasets to spaces that can linearly separate and classify them [15, 16]. Transfer process of input data to the property plane is shown in Figure 4.

Theoretically, any linearly separable SVM can be correctly classified. For a linearly separable dataset, there are *n* samples of training data with two classes expressed as

(2) $$\begin{array}{c} \left(x_{1},y_{1}\right),\ldots \ldots \ldots ,\left(x_{n},y_{n}\right),\;x\in {R\;}^{\;d},y\in \left\{1,-1\right\}. \end{array}$$

These data can be separated from each other by the separator function given by

(3) $$\begin{array}{c} D\left(x\right)=\left(w\;\times \;x\right)+w_{0}. \end{array}$$

The following equations are used for correct classification:

(4) $$\begin{array}{c} \left(w\times x_{i}\right)+w_{0}\geq +1,\;y_{i}=+1, \end{array}$$

(5) $$\begin{array}{c} \left(w\times x_{i}\right)+w_{0}\leq -1,\;y_{i}=-1. \end{array}$$

The appropriate values of *w* and *w*_0_ are calculated to find the optimal separator hyperplane. For real-world data, data samples cannot be distinguished linearly. For this reason, defining a feature mapping function is needed. This function is called the kernel function. The basic idea of kernel methods is to use nonlinear mapping on the input plane first and then apply a linear algorithm to the new input. The training phase is the *K* kernel function of the data in this plane:

(6) $$\begin{array}{c} K=\varphi \left(x_{i}\right)\cdot \varphi \left(x_{j}\right), \end{array}$$

which will depend on the inner products. Decision function is

(7) $$\begin{array}{c} f\left(x\right)=\overset{\text{ls}}{\underset{i=1}{\sum }}\alpha_{i}y_{i}\varphi \left(x_{i}\right)\varphi \left(x\right)+b=\overset{\text{ls}}{\underset{i=1}{\sum }}\alpha_{i}y_{i}K\left(x_{i},x\right)+b\text{.} \end{array}$$

In this function, *α*_*i*_ values are positive Lagrange multipliers. ls is the number of support vectors, and *x*_*i*_ is the support vector [17].

Polynomial kernel, sigmoid kernel, and Gaussian kernel functions are used commonly to find the optimal hyperplane to distinguish linearly nonseparable data.

- Gaussian kernel:

(8) $$\begin{array}{c} K\left(x_{i},x_{j}\right)=\exp\left(-\frac{{\left\|x-y\right\|}^{2}}{\varphi^{2}}\right),\\ K\left(x_{i},x_{j}\right)=e^{-\left\|x^{2}\right\|}e^{-\left\|y^{2}\right\|}e^{2xyT},\\ K\left(x_{i},x_{j}\right)=e^{-\left\|x^{2}\right\|}e^{-\left\|y^{2}\right\|}\overset{\infty }{\underset{n=0}{\sum }}\frac{2^{n}}{n!}{\left(xy^{T}\right)}^{n}\text{.} \end{array}$$

(ii) Polynomial kernel:

(9) $$\begin{array}{c} K\left(x_{i},x_{j}\right)={\left(x_{i}\cdot x_{j}+a\right)}^{b}. \end{array}$$

 
*xy* is the dot product of *x* and *y*. *n*th order of this product is a polynomial kernel. The infinite totaled expression containing all polynomial kernel from the 0th to the infinite order is Gaussian kernel. Therefore, Gaussian is a special kernel and shows good performance.

In order to classify with SVM, the first thing to do is to select a kernel function and related parameters that allow linear separation of the data. For classification of data, the following equation is obtained:

(10) $$\begin{array}{c} K_{ij}=y_{i}y_{j}\varphi \left(x_{i}\right)\cdot \varphi \left(x_{j}\right). \end{array}$$

Appropriate *C* value parameter is chosen and *α* is found:

(11) $$\begin{array}{c} \begin{array}{cc} \max & \left[\overset{L}{\underset{i=1}{\sum }}\alpha_{i}-\frac{1}{2}\alpha^{T}K_{\alpha }\right]\\ \text{s.t} & \begin{array}{c} 0\leq \alpha_{i}\leq C\\ \overset{L}{\underset{i=1}{\sum }}\alpha_{i}y_{i}=0. \end{array} \end{array}\\ \omega =\overset{L}{\underset{i=1}{\sum }}\alpha_{i}y_{i}\varphi \left(x_{i}\right). \end{array}$$

Provided the 0 ≤ *α*_*i*_ ≤ *C* condition, support vectors *V* are determined:

(12) $$\begin{array}{c} b=\frac{1}{N_{v}}\underset{v\in V}{\sum }\left(y_{v}-\underset{i\in V}{\sum }\alpha_{i}y_{i}\varphi \left(x_{i}\right)\varphi \left(x_{v}\right)\right),\\ y^{\prime }=\mathrm{sgn}\left(\omega \varphi \left(x^{\prime }\right)+b\right). \end{array}$$

### 3.4. Particle Swarm Optimization (PSO)

Optimization is the process of achieving the best solution for a problem. Since the methods used in optimization problems defined by mathematical functions are not flexible and the desired result cannot be achieved, new methods have been developed with reference to natural phenomena and PSO is the most common of these algorithms. Inspired by fish and insects moving in flocks, Kennedy and Dr. Eberhart developed PSO in 1995 [18, 19]. It has been shown that the random movements of animals that move in flocks to meet their vital needs are influenced by the other members of the flock and are easier to reach for the purpose of the flock. This process is done for determining the location of the particle with the best position in the stream and the other particles to move in that direction. The particles aim to improve their next position based on their past experience and the individual with the best position in the pack. The PSO algorithm is an evolutionary algorithm like genetic algorithm (GA). However, PSO is faster than GA because there are no operators such as crossover and mutation.

The basic PSO algorithm: every individual in the swarm can be a solution, and every individual is represented by the dimension vector:

(13) $$\begin{array}{c} x_{i}=\left(x_{i1},x_{i2},x_{i3},\ldots ,x_{i\;D}\right)\in S. \end{array}$$

The speed of each individual in the herd is randomly generated. Each individual has the same speed as in equation (14):

(14) $$\begin{array}{c} v_{i}=\left(v_{i1},v_{i2},v_{i3},\ldots ,v_{i\;D}\right)\in S. \end{array}$$

The best local and global positions are determined. Here, the position of each individual is defined as follows:

(15) $$\begin{array}{c} p_{i}=\left(p_{i1},p_{i2},p_{i3},\ldots ,p_{i\;D}\right)\in S. \end{array}$$

Each individual in the PSO adjusts its position around the individual to pbest, global, and gbest. The speed and position information of the individuals are given in the following equations:

(16) $$\begin{array}{c} v_{i}^{\left(t+1\right)}=v_{i}^{\left(t\right)}+c_{1}r_{ir1}\;*\;\left({\text{pbest}}_{i}^{\left(t\right)}-x_{i}^{\left(t\right)}\right)+c_{2}r_{i2}\;*\;\left(\text{gbest}-x_{i}^{\left(t\right)}\right), \end{array}$$

(17) $$\begin{array}{c} x_{i}^{\left(t+1\right)}=x_{i}^{\left(t\right)}+v_{i}^{t+1},\;i=1,\ldots ,P. \end{array}$$

**Figure 5:**
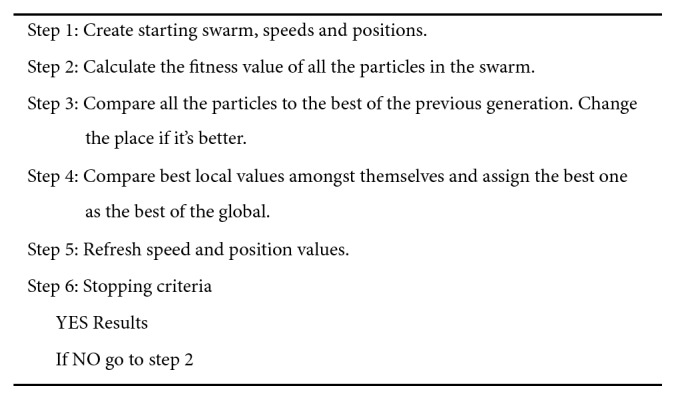
The pseudocode of the PSO [20].

Here, *c*_1_ and *c*_2_ are two social and cognitive acceleration parameters. *r*_1_ and *r*_2_ are random numbers between [0, 1].  Figure 5 shows the pseudocode of the PSO.

### 3.5. Cuckoo Search Algorithm (CS)

CS is a next-generation optimization method based on the hatching parasitic nature of cuckoo birds [21–23]. The most effective features that separate cuckoos from other birds and subject them to the optimization algorithm are aggressive breeding strategies. If the host bird finds that the eggs are not its own, it shows the behavior of throwing the egg from the nest or abandoning the nest. If the eggs are not recognized, the host bird sits on these eggs and the condition of brood parasitism arises. In the CS algorithm, each egg represents a new solution, while each solution is a cuckoo egg. The purpose of this is to use better new solutions to replace the existing poor solution in the nest. As with any optimization problem, CS also has some restrictions that are each cuckoo can leave only one egg in a randomly selected nest and if the nest has a high-quality egg, then the egg is transferred to future generations.

Dropped eggs can be familiarized by the host with a probability of *P*_a_. The probability of *P*_a_ changes in the range [0, 1], and the number of host nests is fixed. Heuristic optimization algorithms perform global and local searches while approaching the best solution. The CS algorithm is an algorithm that is used in combination with global random walk and local random walk approaches.

The global random walk performed with a Levy flight is performed with equation (18). Here, the value produced by the Levy flight is weighted by the variable *α* and is summed to its old position. Thus, new locations are found. *s* and *λ* are control parameters:

(18) $$\begin{array}{c} x_{i}^{t+1}=x_{i}^{t}+\alpha L\left(s,\lambda \right). \end{array}$$

Local random walk is performed with equation (19). Here, *x*_*j*_^*t*^ and *x*_*k*_^*t*^ are the random permutations, *H*(*u*) the Heaviside function, *s* the step length, and *α* a random real number from the Gaussian distribution:

(19) $$\begin{array}{c} x_{i}^{t+1}=x_{i}^{t}+\alpha s⊗H\left(P_{\text{a}}-\in \right)⊗\left(x_{j}^{t}-x_{k}^{t}\right). \end{array}$$

## 4. LabVIEW

The emerging technology needed the development of object-oriented programming languages instead of text-based programming languages. Thus, visual programming was possible without writing code. With National Instruments' development of the LabVIEW program, it was possible to program the model graphically with ready-made functions, and there was no need to write code. With LabVIEW (Laboratory Virtual Instrument Engineering Workbench), it was possible to make programs more quickly and to avoid time loss. LabVIEW generally uses a data flow model instead of text codes. Also, LabVIEW has an ability of multiple parallel processes [24].

**Figure 6:**
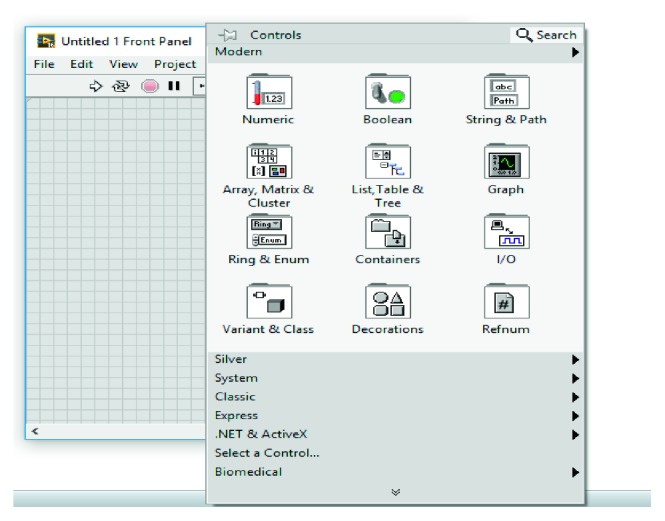
LabVIEW front panel.

**Figure 7:**
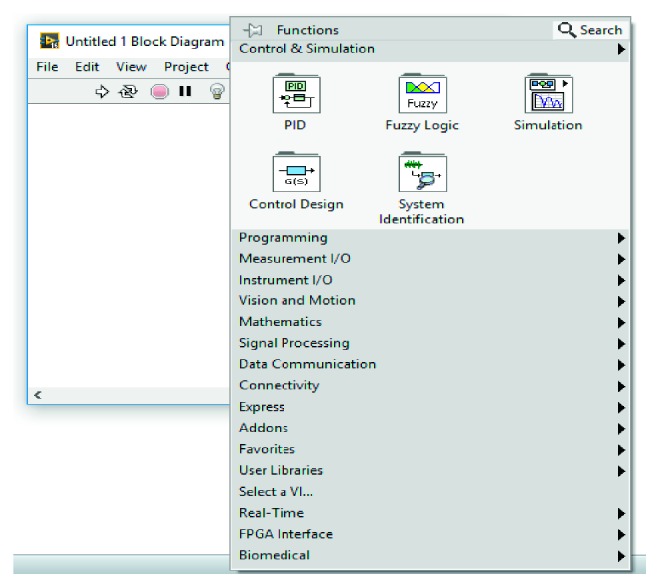
LabVIEW block diagram.

LabVIEW consists of two components: the first one is the front panel that is the user interface and the second one is the block diagram in which graphical codes are shown. Both of them are shown in Figures 6 and 7, respectively. Inputs connected to the virtual instrument on the front panel are called controls, while the outputs are called indicator. The control palette is used in the front panel, and the function palette is used in the block diagram. The control palette allows access to various controls and indicators and is displayed only on the front panel. In the same way, the function palette also allows access to blocks with various functions to design a system and is displayed only in the block diagram. With LabVIEW, subVI can be created just like a VI. Also, a subVI can be created from code already within another VI. The created subVI, with the customized icon and the configured terminals, is used within other VIs repeatedly. The subVI prevents the program from appearing too crowded. As a matter of fact, subVI was used in this study.

## 5. The Proposed Methods

**Figure 8:**
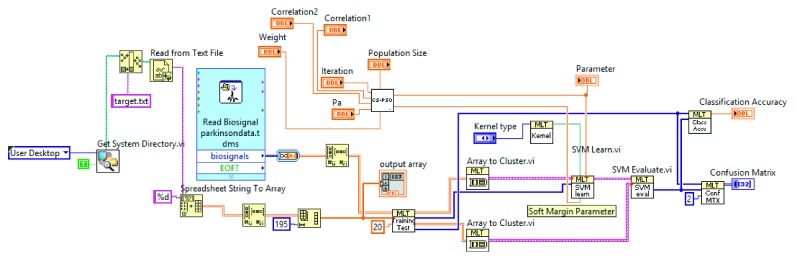
The created hybrid program.

In this study, PSO-SVM, CS-SVM, and CS-PSO-SVM methods are compared with each other. The created hybrid program in the LabVIEW environment is shown in Figure 8. Optimization algorithms are used to find the best SVM parameters. To get these parameters, subVI is created for each optimization method. subVI is frequently used in LabVIEW such as in other programming languages. With subVI, the created program is simplified and is prevented from appearing crowded.

The classification performance results obtained by using SVM parameters determined by optimization are measured by accuracy, precision, recall, *F*1 score, false positive rate (FPR), false discovery rate (FDR), false negative rate (FNR), negative predictive value (NPV), and Matthews' correlation coefficient (MCC) parameters. These parameters are obtained by the confusion matrix. A confusion matrix is shown in Table 2.

### 5.1. Accuracy

Accuracy is the correct classification ratio:

(20) $$\begin{array}{c} \text{accuracy}=\frac{\left(\text{TP}+\text{TN}\right)}{\left(\text{TP}+\text{TN}+\text{FP}+\text{FN}\right)}. \end{array}$$

### 5.2. Precision

Precision is a situation that shows success in a positively predicted situation:

(21) $$\begin{array}{c} \text{precision}=\frac{\text{TP}}{\left(\text{TP}+\text{FP}\right)}. \end{array}$$

### 5.3. Recall

Recall shows how well the positive cases are estimated:

(22) $$\begin{array}{c} \text{recall}=\frac{\text{TP}}{\left(\text{TP}+\text{FN}\right)}. \end{array}$$

### 5.4. *F*1 Score

*F*1 score is the harmonic average of precision and recall:

(23) $$\begin{array}{c} F1\text{ score}=\frac{2\left(\text{precision}*\text{recall}\right)}{\left(\text{precision}+\text{recall}\right)}. \end{array}$$

### 5.5. False Positive Rate (FPR)

FPR, sometimes called the fall-out, is the ratio of misclassified events (FP) to all actual negative events:

(24) $$\begin{array}{c} \text{FPR}=\;\frac{\text{FP}}{\left(\text{FP}+\text{TN}\right)}. \end{array}$$

### 5.6. False Discovery Rate (FDR)

FDR is the expected percent of false predictions in a set of predictions:

(25) $$\begin{array}{c} \text{FDR}=\frac{\text{FP}}{\left(\text{FP}+\text{TP}\right)}. \end{array}$$

### 5.7. False Negative Rate (FNR)

FNR, sometimes called the miss rate, is the proportion of individuals with a known positive condition for which the test result is negative:

(26) $$\begin{array}{c} \text{FNR}=\frac{\text{FN}}{\left(\text{FN}+\text{TP}\right)}. \end{array}$$

### 5.8. Negative Predictive Value (NPV)

NPV is the proportion of individuals with a negative test result for which the true condition is negative:

(27) $$\begin{array}{c} \text{NPV}=\frac{\text{TN}}{\left(\text{TN}+\text{FN}\right)}. \end{array}$$

### 5.9. Matthews' Correlation Coefficient

MCC is a reliable metric used to assess the quality of binary classifiers by taking into account TP, TN, FN, and FP. In fact, MCC is a correlation coefficient between the actual and predictor labels. This parameter takes a value between −1 and +1. The +1 coefficient means an excellent estimate, 0 indicates that the classifier is not better than random estimates, and −1 means a discrepancy between the actual and predicted values [25]:

(28) $$\begin{array}{c} \text{MCC}=\frac{\left(\text{TP}\times \text{TN}\right)-\left(\text{FP}\times \text{FN}\right)}{\sqrt{\left(\text{TP}+\text{FP}\right)\left(\text{TP}+\text{FN}\right)\left(\text{TN}+\text{FP}\right)\left(\text{TN}+\text{FN}\right)}}. \end{array}$$

## 6. Experimental Results

The dataset used in the study is obtained from [4] and has 195 instances and 22 attributes. It is composed of a range of biomedical voice measurements from 31 people. The dataset information is listed in Table 3.  Table 4 lists the appropriate values of the parameters for PSO-SVM, CS-SVM, and CS-PSO-SVM, and the comparison of the models is given in Table 5.

The *P*_a_ parameter in Table 4 is usually selected in the range [0, 1]. In this study, the program was run for different *P*_a_ parameters, and more successful results were obtained for *P*_a_ = 0.262.

As can be seen in Table 5, the performance of the proposed hybrid model is more superior to that of others.

**Figure 9:**
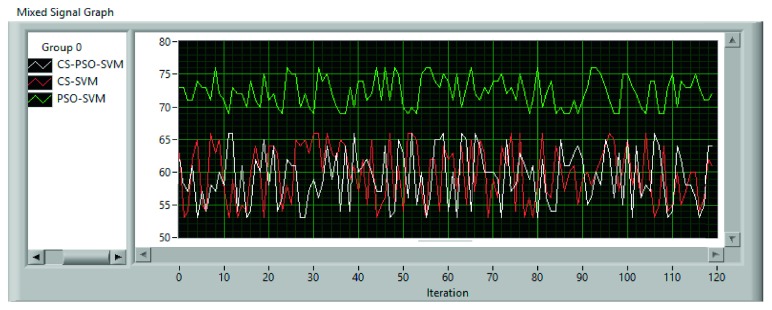
Population average fitness value.

**Figure 10:**
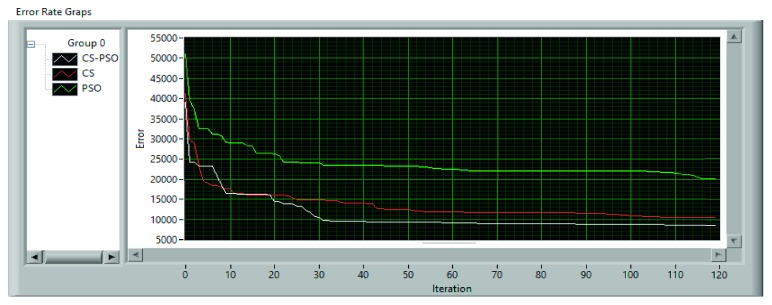
Error rate of each method.

The population average fitness value for the used dataset is shown in Figure 9 for each method. Also, error rates are shown in Figure 10.

## 7. Discussion and Conclusion

Accurate and reliable diagnosis is very important for human health. Different optimization algorithms have been used for optimizing the SVM parameters in this paper. The aim of this paper is to find the best SVM parameters with the hybrid CS-PSO optimization method and obtain best classification accuracy. For this, to analyze the performances of the used methods, the programs were run several times, and the results are presented as tables.  Table 4 shows the appropriate algorithm parameters of different methods used for classification. The performances of the used models are shown in Table 5, and the results show that the best result is obtained by the hybrid model (CS-PSO).

The proposed model achieves a classification accuracy of 97.4359%, while this rate is 92.3077% in CS-SVM and 82.05% in PSO-SVM. The MCC contains all parameters in the confusion matrix. The higher value of MCC proves that the proposed classification method is successful. As shown in Table 5, the highest MCC value was obtained with the proposed hybrid algorithm.

As results of this study, hybrid models created by combining the good characteristics of different optimization algorithms can be used to find the parameters of the classification methods, and the success rate of the model can be increased.

In an increasingly widespread LabVIEW environment, it is possible to quickly create subprograms and to obtain results quickly.

## Figures and Tables

**Table 1:** Attributes of used data.

Number	Attributes	Information
1	MDVP:Fo (Hz)	Average vocal fundamental frequency
2	MDVP:Fhi (Hz)	Maximum vocal fundamental frequency
3	MDVP:Flo (Hz)	Minimum vocal fundamental frequency
4	MDVP:Jitter (%)	Several measures of variation in the fundamental frequency
5	MDVP:Jitter (Abs)	
6	MDVP:RAP	
7	MDVP:PPQ	
8	Jitter:DDP	
9	MDVP:Shimmer	Several measures of variation in amplitude
10	MDVP:Shimmer (dB)	
11	Shimmer:APQ3	
12	Shimmer:APQ5	
13	MDVP:APQ	
14	Shimmer:DDA	
15	NHR	Two measures of ratio of noise-to-tonal components in the voice status
16	HNR	
17	RPDE	Two nonlinear dynamic complexity measures
18	D2	
19	DFA	Signal fractal scaling exponent
20	Spread1	Three nonlinear measures of the fundamental frequency variation
21	Spread2	
22	PPE	

**Table 2:** Confusion matrix.

Prediction	Actual	
	Positive	Negative
Positive	TP	FP
Negative	FN	TN

**Table 3:** Dataset information.

Number of instances	Number of attributes	Normal	PD
195	22	8	23

**Table 4:** Parameter settings.

Method	Population size	Iteration	*c*_1_	*c*_2_
PSO-SVM	18	120	1.3	1.87
CS-SVM (*P*_a_ = 0.262)	18	120	—	—
CS-PSO-SVM (*P*_a_ = 0.262)	18	120	1.3	1.87

**Table 5:** Obtained results.

Method	Accuracy (%)	Precision (%)	Recall (%)	*F*1 measure (%)	FPR	FDR	FNR	NPV	MCC
PSO-SVM	82.05	88.89	57.14	69.57	0.04	0.1111	0.4286	0.80	0.6051
CS-SVM	92.3077	83.33	90.91	86.96	0.0714	0.1667	0.0909	0.9630	0.8167
CS-PSO-SVM (*P*_a_ = 0.262)	97.4359	100	90.91	95.24	0	0	0.0909	0.9655	0.9369

## Data Availability

The data that support the findings of this study are available from the authors upon reasonable request.
